# IBD prevalence in Lothian, Scotland, derived by capture–recapture methodology

**DOI:** 10.1136/gutjnl-2019-318936

**Published:** 2019-07-11

**Authors:** Gareth-Rhys Jones, Mathew Lyons, Nikolas Plevris, Philip W Jenkinson, Cathy Bisset, Christopher Burgess, Shahida Din, James Fulforth, Paul Henderson, Gwo-Tzer Ho, Kathryn Kirkwood, Colin Noble, Alan G Shand, David C Wilson, Ian DR Arnott, Charlie W Lees

**Affiliations:** 1 Centre for Inflammation Research, The Queen’s Medical Research Institute, University of Edinburgh, Edinburgh, UK; 2 Edinburgh IBD Unit, Western General Hospital, Royal Victoria Building, Edinburgh, UK; 3 Child Life and Health, University of Edinburgh, Edinburgh, UK; 4 Department of Paediatric Gastroenterology and Nutrition, Royal Hospital for Children and Young People, Edinburgh, UK; 5 Histopathology Unit, Western General Hospital, Royal Victoria Building, Edinburgh, UK

**Keywords:** epidemiology, inflammatory bowel disease, Crohn’s disease, ulcerative colitis

## Abstract

**Objective:**

IBD prevalence is estimated to be rising, but no detailed, recent UK data are available. The last reported prevalence estimate in the UK was 0.40% in 2003. We aimed to establish the current, and project future, prevalence in Lothian, Scotland.

**Design:**

We conducted an all-age multiparameter search strategy using inpatient IBD international classification of disease (ICD-10) coding (K50/51)(1997–2018), IBD pathology coding (1990–2018), primary and secondary care prescribing data (2009–2018) and a paediatric registry, (1997–2018) to identify ‘possible’ IBD cases up to 31/08/2018. Diagnoses were manually confirmed through electronic health record review as per Lennard-Jones/Porto criteria. Autoregressive integrated moving average (ARIMA) regression was applied to forecast prevalence to 01/08/2028.

**Results:**

In total, 24 601 possible IBD cases were identified of which 10 499 were true positives. The point prevalence for IBD in Lothian on 31/08/2018 was 784/100 000 (UC 432/100 000, Crohn’s disease 284/100 000 and IBD unclassified (IBDU) 68/100 000). Capture–recapture methods identified an additional 427 ‘missed’ cases (95% CI 383 to 477) resulting in a ‘true’ prevalence of 832/100 000 (95% CI 827 to 837).

Prevalence increased by 4.3% per year between 2008 and 2018 (95% CI +3.7 to +4.9%, p<0.0001). ARIMA modelling projected a point prevalence on 01/08/2028 of 1.02% (95% CI 0.97% to 1.07%) that will affect an estimated 1.53% (95% CI 1.37% to 1.69%) of those >80 years of age.

**Conclusions:**

We report a rigorously validated IBD cohort with all-age point prevalence on 31/08/2018 of 1 in 125, one of the highest worldwide.

**Significance of this study**

**What is already known on this subject?**

- IBD incidence appears to be stabilising in Western populations though UK data has not been reported for >15 years, when prevalence was reported at 0.4%.
- Recent nationwide data from Canada estimate 2018 prevalence at 0.7% and introduce the phenomenon of compounding prevalence in IBD.

**What are the new findings?**

- The point prevalence on 31/08/2018 for IBD in Lothian, Scotland, is 1 in 125 (0.8%).
- Incidence has been static in the last decade, but prevalence rose by 4.3% per year between 2008 and 2018.
- Due to incidence exceeding mortality, prevalence will continue to rise (compounding prevalence). The projected prevalence in 2028 for IBD in Lothian is 1 in 98 (1.0%).
- In 2028, the number of IBD patients >40 years of age will exceed the entire current IBD population. Moreover, the majority of patients with IBD in 2028 will be >50 years old.

**How might it impact on clinical practice in the foreseeable future?**

- These data provide current and projected prevalence rates that are of immediate utility to healthcare providers, resource planning, professional bodies and patient groups.

## Introduction

The prevalence of UC, Crohn’s disease (CD) and colonic IBD, type unclassified (IBDU), collectively termed IBD, in the Western world is reported to be increasing.^1^ Overall IBD prevalence is estimated at >0.3% in a recent review, though there is considerable geographical variation within this.^2^

The current prevalence of IBD in the UK is poorly described. Although there have been four studies of UK adult IBD prevalence included in a recent systematic review, no UK data are available for the past 15 years.^2^ Therefore, the most recent estimates of UK IBD prevalence are 9–144/100 000 for CD and 66–389/100 000 for UC.^3 4^

While IBD prevalence increases in Westernised countries, incidence is seemingly static in these populations.^2^ In contrast to pre-1990 data, where 75% of CD and 60% of UC studies reported a rising incidence, 73% and 83% of post-1990 studies show a stable or falling incidence for CD and UC, respectively.^2^ Therefore, while IBD continues to be incurable, with significant morbidity but low mortality, prevalence will continue to increase. This phenomenon of compound prevalence will likely affect global IBD epidemiology, as life expectancy increases worldwide, but arguably will be greatest in developing countries that also have rising incidence.^1^ This will likely have dramatic effects on the number of elderly patients with IBD in the future, a cohort that poses significant challenges in delay in diagnosis, comorbidity and polypharmacy.^5^ However, understanding the rate of change in elderly IBD prevalence is currently poorly described.

Scotland is an attractive nation to perform population-based studies. Healthcare is universal and standardised with excellent national registries covering >99% of the population for birth, mortality and cancer, with individual patient linkage afforded by a unique community health index number (https://www.ndc.scot.nhs.uk/Dictionary-A-Z/Definitions/index.asp?Search=C&ID=128&Title=CHI%20Number). Capture–recapture is a method for estimating the number of individuals in a closed population, originally developed by naturalists to estimate species number by sampling, marking and releasing animals over numerous time points. The same approach can be used in humans to estimate prevalence by comparing multiple, independent datasets to ‘capture’ individuals, using statistical modelling to infer the number of ‘missed’ cases.^6^

NHS Lothian Health Board provides all primary and secondary care to its residents and serves 16.5% of the Scottish population (as of 2018). We aimed to perform an IBD cohort study of incidence and prevalence over the past 10 years using capture–recapture methods to identify those information streams that most accurately identified true cases. We then sought to report point prevalence on 31/08/2018 and project IBD prevalence in 2028.

## Methods

### Study population and case identification

Scotland has a population of 5.45 million people (National Records for Scotland (NRS) 2016-based 2018 projection, https://www.nrscotland.gov.uk/statistics-and-data/statistics/statistics-by-theme/population), is located at 56.49^o^N 4.20^o^W, with a total land area of 77 933 km^2^. NHS Lothian provides all healthcare for a geographically well-defined 897 210 people (NRS 2018 projection) in Edinburgh and the surrounding area, amounting to the second largest residential population in Scotland. Four hospitals serve this area (Royal Infirmary of Edinburgh (RIE), Western General Hospital (WGH), St John’s Hospital (SJH) and the Royal Hospital for Sick Children, Edinburgh (RHSCE)).

We sought to describe the point prevalence of IBD on 31/08/2018 in Lothian, Scotland. Census data are performed every 10 years (most recently in 2011) with NRS reporting yearly, mid-year (June) population estimates published retrospectively the following April. Mid-2018 population projections were used for 31/08/2018 point prevalence reporting, based on 2016 estimates, as these were the most current data at time of submission.

**Figure 1:**
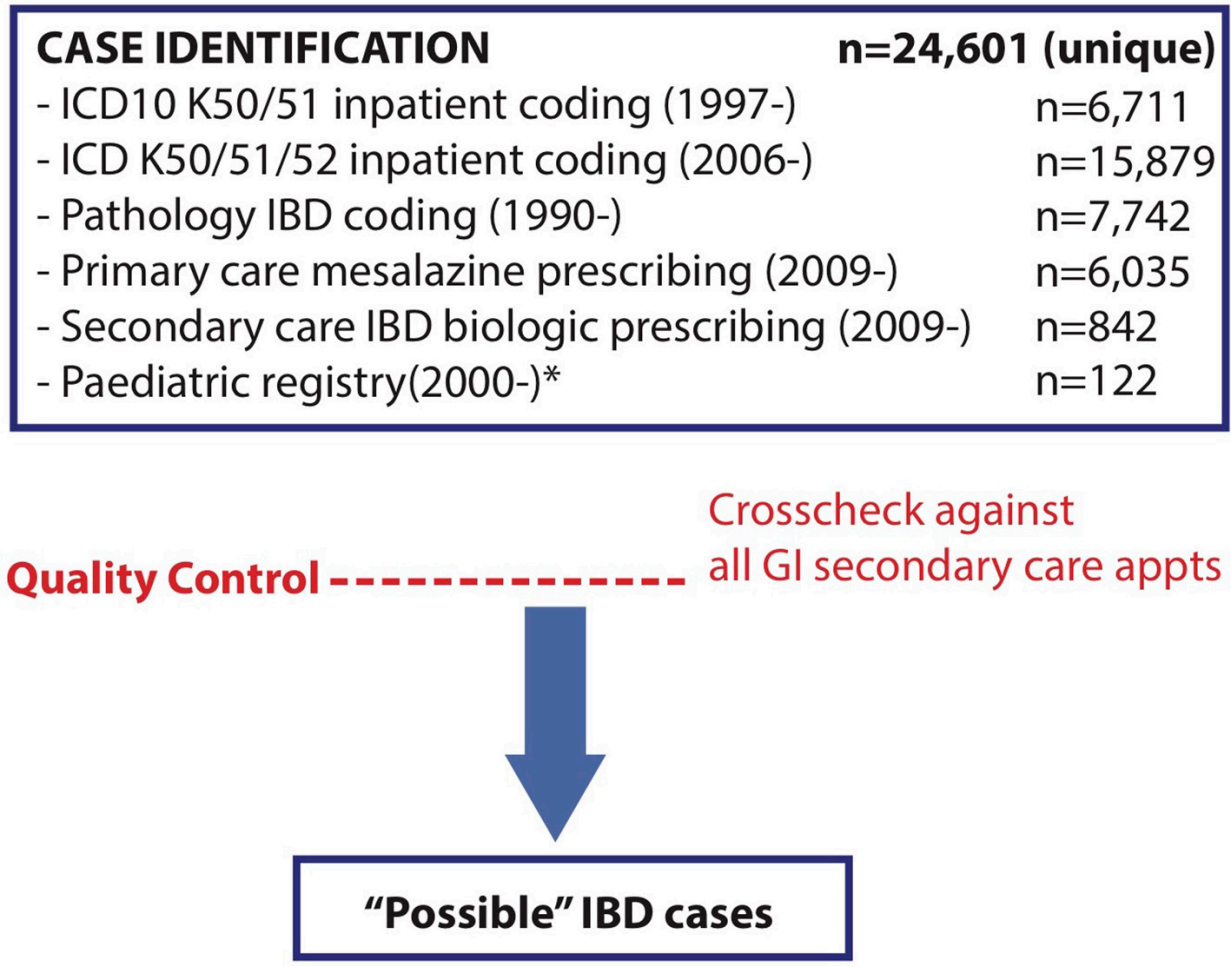
Information sources used for IBD case identification. The number of unique cases of putative IBD identified from each information stream, and overall, during the described capture period. All gastroenterology secondary care outpatient appointments between 01/08/2017 and 01/08/2018 at the largest Lothian IBD centre (Western General Hospital) were screened in addition to estimate accuracy of our search strategy. All ‘possible’ cases were then submitted to manual review of the electronic medical record.

‘Possible’ IBD cases were identified using the six information sources detailed in figure 1 and subjected to manual case verification using protocolised review of the electronic health record (EHR) and Lennard-Jones/Porto criteria by a team of eight IBD physicians. The EHR contains all secondary care interactions since 2009 using the TrakCare (InterSystems Corporation, Cambridge, Massachusetts, USA) system across all Lothian sites. Where diagnosis was not clear, an expert panel of three IBD physicians reviewed the cases. All six information streams capture the entire Lothian population, but over different time periods, as discussed below.

Histopathology processing and analysis for IBD was historically performed at two sites (RIE and WGH) for the whole of NHS Lothian, and a single site (WGH) for the last 10 years. Tissue samples have been coded prospectively for diagnoses since 1988 but recorded electronically since 1990. The pathology database was interrogated for IBD cases from 01/01/1990 to 31/8/2018 by a consultant IBD histopathologist.

All hospital admissions in NHS Lothian have been coded within TrakCare since 01/08/2006 using international classification of disease (ICD-10) criteria. K50 (Crohn’s disease), K51 (UC) and K52 (Other unspecified non-infective gastroenteritis and colitis) codes were obtained where they appeared anywhere within the discharge summary. We included all K52 codes (K52.3 is IBDU) to ensure IBD/IBDU cases that were incorrectly coded or where the diagnosis was ultimately changed, were also captured. Information Services Division Scotland also collects data on K50/51 admission codes as part of routine data capture for the Scottish government that predates the EHR system to 01/01/1997. In-patient K50/51 coding data were therefore obtained from 01/01/1997 to 31/03/2018, with K52 coding available in addition from 01/08/2006.

Primary care prescribing in Lothian is standardised using a joint formulary and complete for all prescribed drugs from 01/04/2009. Due to requisite monitoring of immunomodulator drugs, all IBD patients prescribed these medications are seen in secondary care and therefore identified from the specialist clinic attendance (see below). We hypothesised that IBD patients with a benign natural history may not have been captured from other information streams (e.g. no hospital admission, pre-1990 or no recorded pathology). We therefore assessed mesalazine prescribing (British National formulary section 5.1) in primary care from 01/04/2009 to 31/03/2018.

Prescribing of biological agents in IBD (infliximab, adalimumab, ustekinumab, vedolizumab) in Lothian is exclusively performed in secondary care and requires regular monitoring including yearly secondary care review and bi-annual virtual biologic clinic review. A prospective adult Lothian biological database in IBD has been held since 01/08/2009; all entries were therefore submitted for case verification.

Outpatient clinic attendances in secondary care are recorded electronically on the EHR, but not universally coded using ICD-10 (RIE but not SJH, WGH or RHSC), in contrast to the inpatient admission data. We therefore screened all GI clinic attendances from the largest hospital that does not code outpatient attendances (WGH) that also provides the vast majority of specialist IBD care, from 01/08/2017 to 31/08/2018, to assess the completeness of our search strategy.

The specialist paediatric IBD service at RHSCE acts as a dedicated central referral centre for all district general hospitals within a strict geographical area of South-East Scotland (based on postcode). Since 1997, all incident and prevalent cases of paediatric IBD managed within South-East Scotland have been prospectively recorded on a custom-made database which is regularly updated to capture patient immigration, emigration, transition to adult services and death, to ensure accurate prevalence figures. All cases are validated according to the revised Porto criteria and no age limitation is placed to ensure full capture of all patients being cared for in paediatric services. All prevalent IBD cases at 31/08/2018 confined to Lothian postcodes and cared for within paediatric services were added to the above identified adult cases.

Incident adult IBD cases between 2008 and 2017 were obtained by documenting the date of diagnosis during the EHR verification process by manual case review of pathology, endoscopy reports where necessary and EHR (that includes all secondary care attendance). Incident cases were reported per calendar year and per 100 000 population calculated using the mid-year population estimates (NRS) for that calendar year.

### Data linkage

All confirmed IBD cases were cross-matched against the national mortality registry, and the EHR for last known postcode (all secondary care patient interactions require address details to be confirmed as being current) to identify prevalent cases. WGH and RIE take tertiary care referrals from other non-Lothian hospitals, therefore, only postcodes exclusive to NHS Lothian Health Board were used to identify prevalent cases.

### Statistical analysis

JMP V.14 (SAS Software, Copyright © (2019) SAS Institute Inc, Cary, North Carolina, USA) and Prism V.7.00 (GraphPad Software, La Jolla, California USA, www.graphpad.com) were used for statistical analyses and generation of graphs. Descriptive statistics are presented as medians with IQR for continuous variables and frequencies with percentages for categorical variables. For comparison of nonparametric continuous variables, the Mann-Whitney U test or Kruskall-Wallis test was used where appropriate. For comparison of categorical variables, the χ^2^ test was used. A p value of <0.05 was considered significant for all statistical tests. Trends in incidence, prevalence and mortality over time were reported as annual percentage change, calculated by exponentiating the beta-coefficient of Poisson regression and subtracting 1. Poisson modelling was also used to calculate significance in these trends over time.

In epidemiology, capture–recapture approaches estimate the degree of incomplete case ascertainment using overlapping case lists from multiple sources. A three-source model using the best performing information sources (termed A, B and C) was employed as this has been shown to be optimal in other diseases.^6^ Loglinear Poisson regression was used to explore the dependence between sources using interaction terms. We thus generated a dataset with four variables (A, B, C and frequency) where A, B and C were equal to 0 or 1 (0=not identified in this source, 1=identified in this source) and frequency determined the number of cases identified from each combination of A, B and C. The goodness of fit for each model combination was assessed using deviance and the derived Bayesian information criterion (BIC) with the lowest BIC model selected. To derive missing case number, the model was used to estimate case frequency when A+B+C=0.

To estimate future prevalence, we used an autoregressive (AR) integrated (I) moving average (MA)(ARIMA) model. This analysis relies on historical values at regular time intervals with the assumption of stationarity to model future values. The ARIMA model is a time series analysis used extensively in economic and prevalence forecasting as it permits analysis at a specific time point relative to previous dependent, equally spaced, discrete data and has been used to forecast IBD prevalence in recent nationwide analysis.^1^ We therefore used standard Box-Jenkins ARIMA methodology to assess the ability of AR or MA modelling after differencing, if required, to forecast future prevalence. Observed prevalence (using prevalent cases from 01/01/2008 to 01/08/2018 at monthly intervals, standardised for the mid-population estimates from that calendar year from NRS data and not including estimated missed cases from capture–recapture methodology) for all IBD and for individual age groups (<17, 17–39, 40–59, 60–79 and >80 years) were imputed to JMP V.14.

First, stationarity of the dataset was assessed. Where stationarity was not observed, differencing was used to achieve stationarity. All the prevalent age groupings and IBD prevalence overall required one mode of differencing to achieve stationarity. Thereafter, AR or MA models were compared for best fit using Akaike Information Criterion (AIC), BIC and Root Mean Square Error (RMSE) for each AR/MA combination. The model with the lowest AIC/BIC and RMSE was used. A differencing of 1 and AR model without intercept produced the best fit for total cohort prevalence. A simulated time lapse using the ARIMA model was then undertaken to 01/08/2028 and reported with 95% prediction intervals. The forecast number of prevalent IBD cases per age group in 2028 were then derived from NRS mid-year population estimates.

### Ethical considerations

Data are reported according to the Strengthening the Reporting of Observational Studies in Epidemiology statements.^7^

## Results

### IBD point prevalence on 31 August 2018

**Figure 2:**
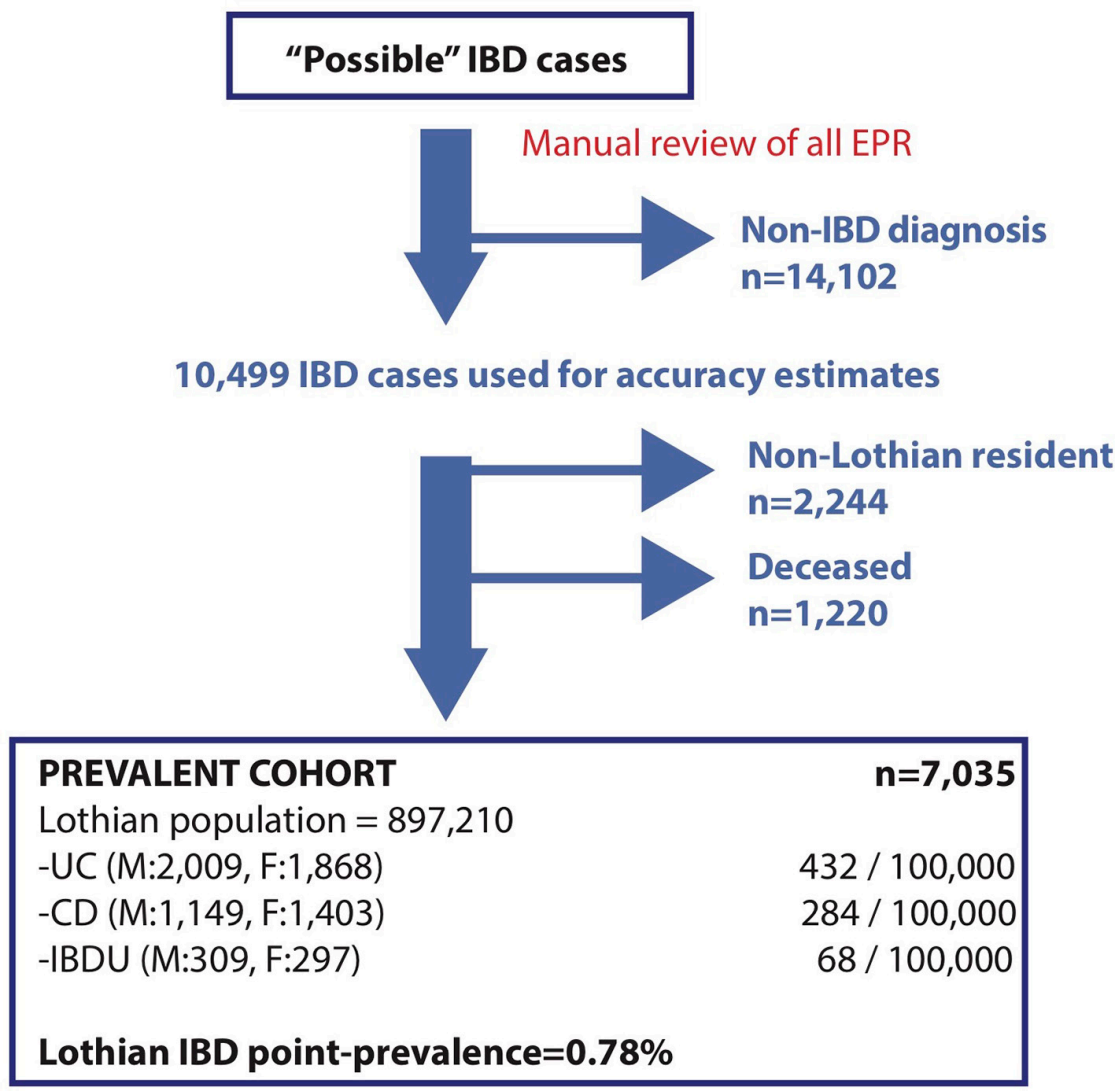
The point prevalence of IBD in Lothian on 31/08/2018. True and false positive cases were identified from ‘possible’ cases in figure 1 and used to define accuracy rates for each information stream (Table 3). True positives were screened for prevalent (Lothian postcode) and live (linkage to national datasets) cases to report point prevalence on 31/08/2018.

There were 7035 prevalent IBD cases in Lothian (2552 CD, 3877 UC and 606 IBDU) (figure 2) conferring a point prevalence on 31/08/2018 of 784/100 000 (284/100 000 for CD, 432/100 000 for UC and 68/100 000 for IBDU) (figure 2 and table 1).

**Table 1:** Overview of the demographic information for prevalent IBD cases in  the Lothian IBD registry on 31/08/2018

Variable	CD (n=2552)	UC (n=3877)	IBDU (n=606)
Female (n (%))	1403 (55.0)	1868 (48.2)	305 (49.0)
Age (Median, IQR)	49.1 (34.8–62.4)	52.6 (39.4–65.9)	51.0 (37.6–63.0)
Age at diagnosis (Median, IQR)	29.4 (21.0–45.6)	36.8 (26.1–50.1)	42.0 (27.6–54.5)
Disease duration (Median, IQR)	11.7 (5.7–20.7)	10.7 (5.4–18.7)	6.9 (3.5–12.2)

CD, Crohn’s disease; IBDU, IBD unclassified.

**Figure 3:**
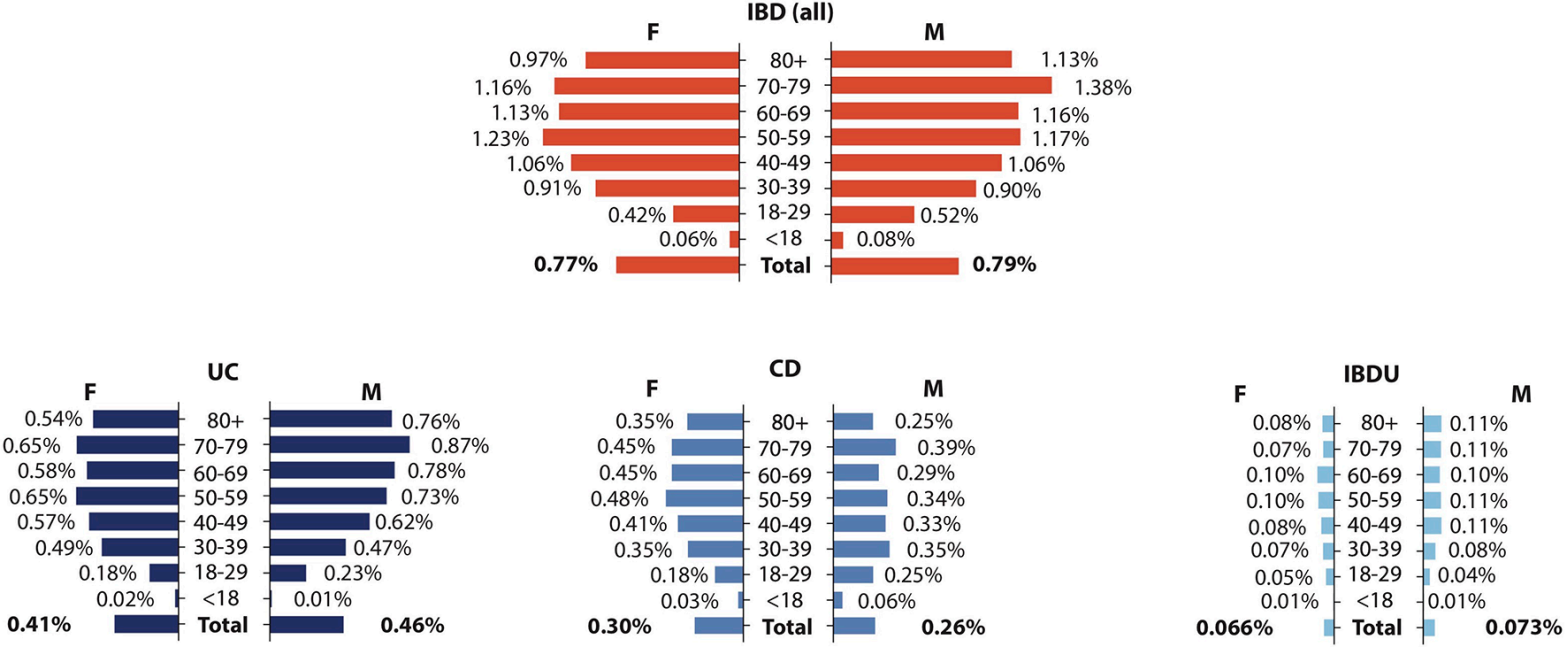
Age group prevalence breakdown by sex and IBD classification. Prevalent IBD cases were subdivided into age groups by IBD diagnosis into all IBD (red) and UC, CD or IBDU (blue). Percentage prevalence is reported for age-appropriate population data derived from National records for Scotland, 2016-based projections for 2018. CD, Crohn’s disease; IBDU, IBD unclassified.

The IBD point prevalence on 31/08/2018 was 776/100 000 (women) and 796/100 000 (men) with prevalence highest in women aged 50–59 and men aged 70–79 (1.24% and 1.38%, respectively, figure 3). There was no association between postcode and IBD prevalence nor incidence (online supplementary figure 1). When stratified by age group, the measured point prevalence on 31/08/2018 was 106/100 000 for <17 years old, 652/100 000 for 17–39 years old, 1124/100 000 for 40–59 years old, 1178/100 000 for 60–79 years old and 1042/100 000 for >80 years old groups (online supplementary table 1).

10.1136/gutjnl-2019-318936.supp1 Supplementary data

10.1136/gutjnl-2019-318936.supp2 Supplementary data

The annual average percentage change in prevalence rose significantly between 2008 and 2018 at 4.3% (95% CI 3.7 to 4.9) per year (p<0.0001)(Table 2). The proportion of IBD subtypes within the prevalent cohort over this time was unchanged, suggesting the prevalence of IBD subtypes was increasing uniformly (online supplementary figure 2).

10.1136/gutjnl-2019-318936.supp3 Supplementary data

**Table 2undefined:** Standardised prevalence per 100 000 population for Lothian on 31 August between 2008 and 2018

Year	Population	Prevalent IBD cases per 100 000 population			
		UC	CD	IBDU	All
2008	808 940	315	216	36	567
2009	816 510	334	224	38	596
2010	825 520	349	232	41	621
2011	836 610	359	242	43	644
2012	843 740	375	249	47	671
2013	849 720	386	258	51	694
2014	858 120	398	265	56	719
2015	867 800	411	270	59	739
2016	880 000	418	274	62	753
2017	889 450	427	278	66	772
2018	897 210	432	284	68	784

CD, Crohn’s disease; IBDU, IBD unclassified.

### Information source accuracy

In total, 24 601 unique possible IBD cases were identified from pathology coding (n=7742), hospital admission coding (K50/51/52 coding (2006–2018) n=15 879, K50/51 coding (1997–2018) n=6711), primary care prescribing of mesalazine (n=6035), IBD biological prescribing (n=842) and paediatric cases (n=122) (Table 3 and online supplementary figure 3).

10.1136/gutjnl-2019-318936.supp4 Supplementary data

**Table 3undefined:** Accuracy of IBD case identification information streams

Data source	True positives n / %	False positives n / %	% of total true positives identified
Pathology coding	7661 (99)	81 (1)	73
ICD-10 code K50/51	5525 (75)	1186 (25)	53
Mesalazine prescribing	5079 (84)	956 (16)	48
ICD-10 code K50/51/52	4254 (27)	11 625 (73)	40
Secondary care prescribing	842 (100)	0	8
Paediatric registry	122 (100)	0	1
**Total unique cases**	10 499	14 102	

All true positive, live Lothian or non-Lothian resident IBD cases identified from manual case review of ‘possible’ cases were used to derive the accuracy of each information stream. The proportion of cases accepted as true positive (ie, IBD) or false positive (ie, non-IBD) for each information stream is presented, in addition to the proportion of all true positives.

Possible IBD cases were screened manually by EHR review, leaving 10 499 true positives (Table 3). Inpatient coding (restricted to K50/51) correctly identified IBD in 75% of cases, with 53% of the cohort hospitalised for any indication since 1997 (Table 3). To identify prevalent cases, postcode and death linkage analysis was performed, with 2244 patients possessing a non-Lothian postcode and 1220 patients deceased as of 31/08/2018, which were thus excluded (figure 2).

The optimum search strategy using the minimum number of data streams was pathology plus K50/51 coding and mesalazine prescribing, which identified 94.6% of true positives (online supplementary figure 3). Without manual EHR review, this strategy would overestimate the number of IBD cases by 6.0%, which increased to 17.0% if pathology was removed from the algorithm (online supplementary figure 4 and Table 3).

10.1136/gutjnl-2019-318936.supp5 Supplementary data

To identify the number of missed cases from our identification process, loglinear Poisson regression was applied to the three best performing information streams (K50/51 coding (A), mesalazine prescription (B) and pathology (C)). Loglinear models were compared for BIC, deviance and R^2^ using multiple interaction terms between information streams to identify the optimum model (A, B, C with AB and AC interaction terms, R^2^=0.98, deviance=17, BIC=93, p<0.001). We therefore estimate that 427 cases (95% CI 383 to 477) have escaped our information capture. When combining observed and missed cases, we estimate the ‘true’ prevalence of IBD to be 832/100 000 (95% CI 827 to 837) and report the completeness of our registry to be 94.3% (95% CI 93.7 to 94.8).

### Incidence, mortality and age at diagnosis

**Figure 4:**
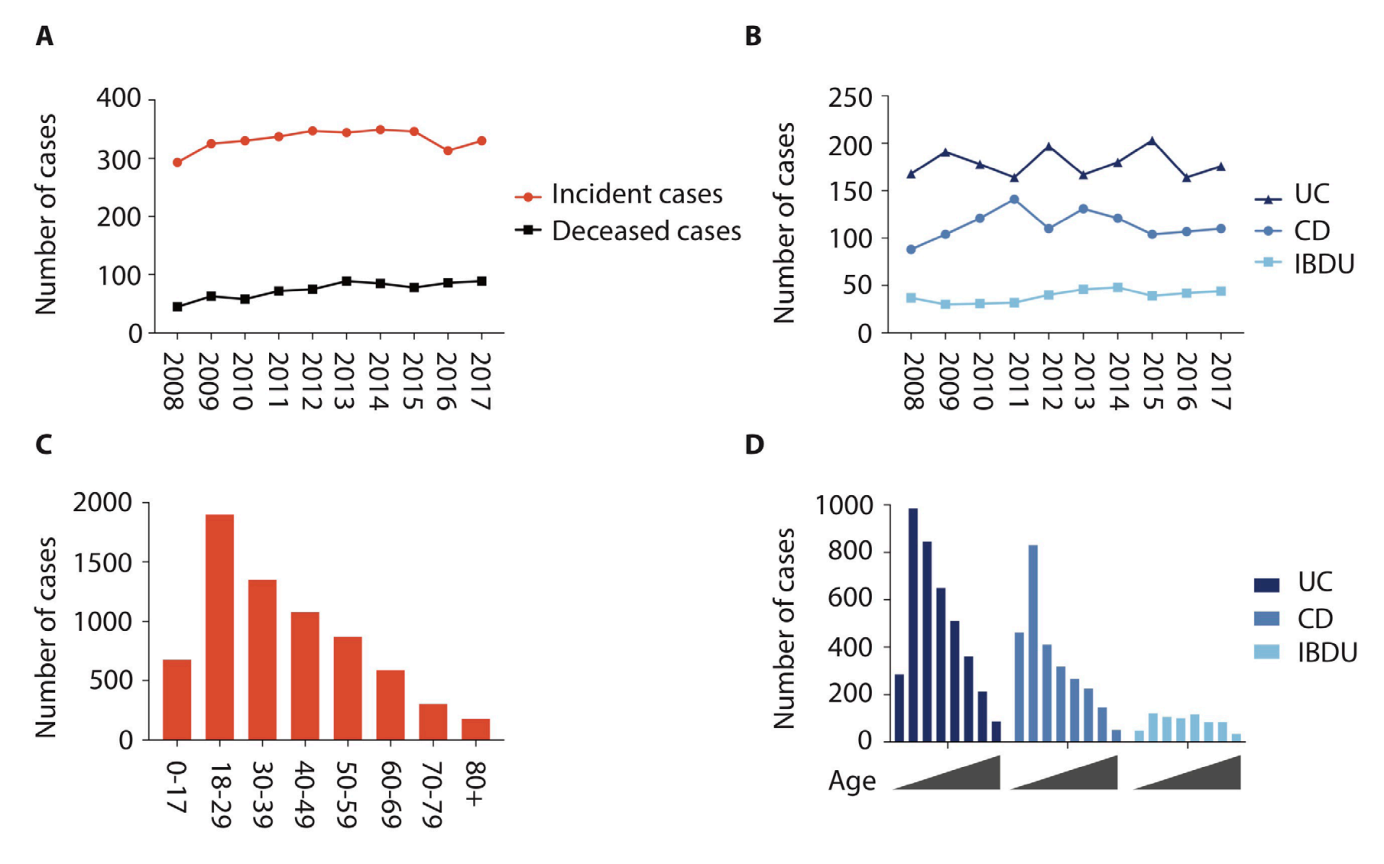
Incidence, mortality and age at diagnosis by IBD subtype changes between 2008 and 2018. Incident cases were identified by using the date of diagnosis obtained during case verification. Mortality data were obtained from national registry linkage. Incidence and prevalent IBD cohort mortality (A), with IBD subtype incidence breakdown (B), age at diagnosis for prevalent cases on 31/08/2018 overall (C) and for IBD subtype (D). CD, Crohn’s disease; IBDU, IBD unclassified.

IBD incidence was 40.8/100 000 patient years in 2017 (19.8/100 000 UC, 13.6/100 000 CD and 5.4/100 000 IBDU) (figure 4A and B and table 4). However, in contrast to prevalence, IBD incidence per calendar year from 2008 to 2017 was static, with no significant change overall (annual average percentage change 14.4%; 95% CI −0.9 to +32.1, p=0.66).

**Table 4undefined:** Incident cases and standardised incidence per 100 000 per calendar year for Lothian between 2008 and 2017

Year	Background population (mid-year estimates)*	UC		CD		IBDU		All	
		Number of cases	Per 100 000 per year	Number of cases	Per 100 000 per year	Number of cases	Per 100 000 per year	Number of cases	Per 100 000 per year
2008	808 940	168	20.77	88	10.88	37	4.57	293	36.22
2009	816 510	191	23.39	104	12.86	30	3.71	325	40.18
2010	825 520	178	21.56	121	14.96	31	3.83	330	40.79
2011	836 610	164	19.60	141	17.43	32	3.96	337	41.66
2012	843 740	197	23.35	110	13.60	40	4.94	347	42.90
2013	849 720	167	19.65	131	16.19	46	5.69	344	42.52
2014	858 120	180	20.98	121	14.96	48	5.93	349	43.14
2015	867 800	203	23.39	104	12.86	39	4.82	346	42.77
2016	880 000	164	18.64	107	13.23	42	5.19	313	38.69
2017	889 450	176	19.79	110	13.60	44	5.44	330	40.79

*Census data are presented for 2011, the remaining population estimates are taken from NRS mid-year (June) population estimates.

CD, Crohn’s disease; IBDU, IBD unclassified; NRS, National Records for Scotland.

Mortality of the prevalent population per calendar year did not change significantly between 2008 and 2017 (annual average percentage change in mortality 10.5%, 95% CI −18.8 to +50.0, p=0.52)(figure 4A). Age at diagnosis peaked in the 18–29 age group in all IBD subtypes and overall, falling thereafter with increasing age in UC and CD, but with an even age distribution in IBDU (figure 4C and D).

### Projected IBD prevalence in 2028

**Figure 5:**
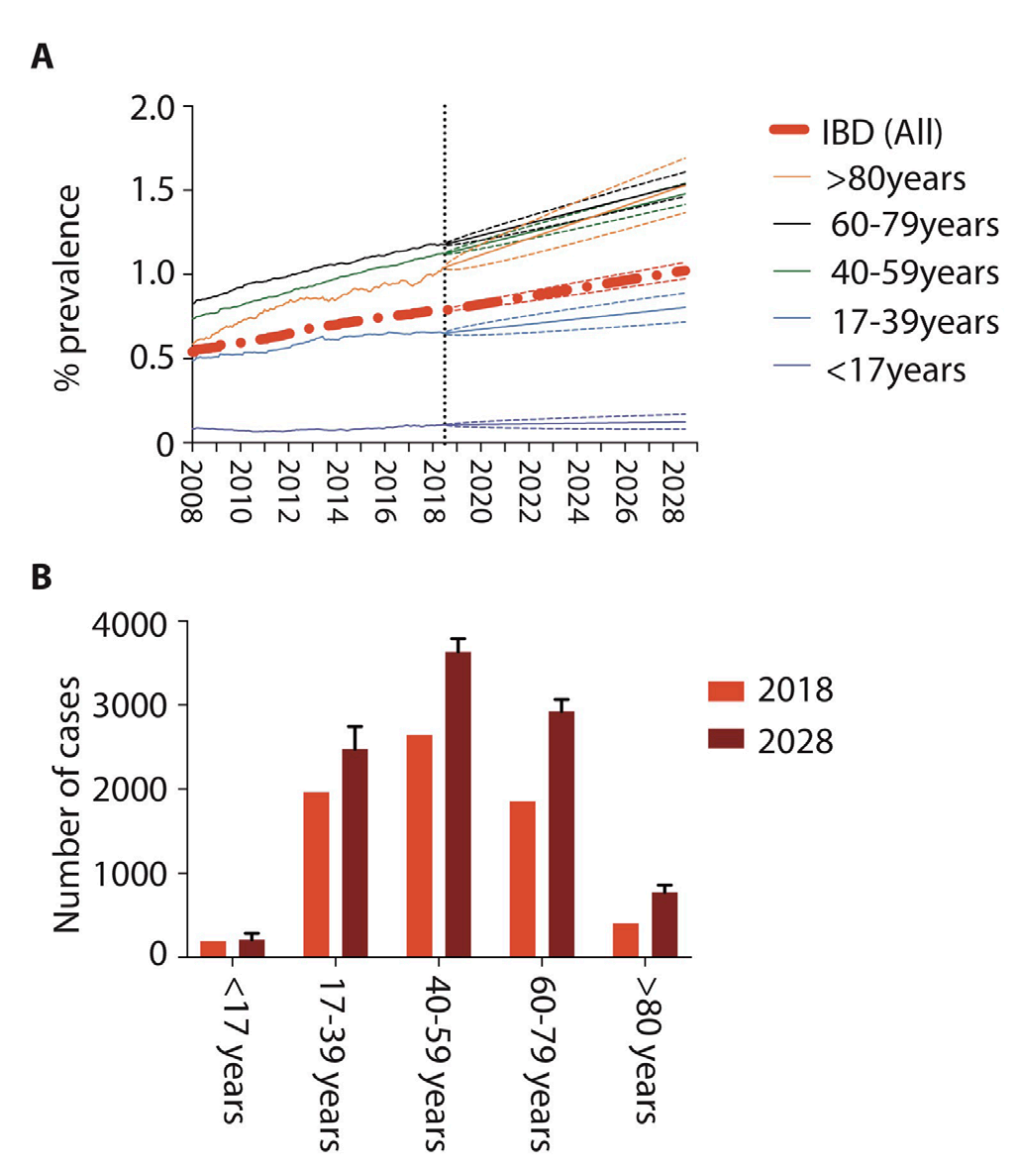
Projected IBD prevalent cases±95% CI from 2018 to 2028. Retrospective prevalence data were imputed monthly from 01/01/2008 to 31/08/2018 to model age-group projected future prevalence to 31/08/2028 (A), with absolute prevalent case number per age group for actual prevalence on 31/08/2018 and projected prevalence on 01/08/2028 (B). 2016-based population projections were obtained from 2018 to 2028 from National records for Scotland data.

The forecast point prevalence for IBD in Lothian on 01/08/2028 is 1023/100 000 (95% CI 975 to 1071), which equates to 9681 people (95% CI 9393 to 10 329) (figure 5A).

When stratified by age group, the estimated point prevalence on 01/08/2028 is 124/100 000 for <17 years old (95% CI 80 to 169), 804/100 000 for 17–39 years old (95% CI 718 to 890), 1479/100 000 for 40–59 years old (95% CI 1416 to 1542), 1537/100 000 for 60–79 years old (95% CI 1464 to 1610) and 1530/100 000 for >80 years old (95% CI 1368 to 1692) groups, respectively (figure 5A).

This equates to 210 aged under 17 (95% CI 135 to 284), 2478 aged 17–39 (95% CI 2213 to 2743), 3635 aged 40–59 (95% CI 3479 to 3790), 2928 aged 60–79 (95% CI 2788 to 3067) and 776 aged >80 (95% CI 694 to 858) patients with IBD in Lothian on 01/08/2028 (figure 5B).

We conducted a secondary analysis of prevalence projections in 2028 using total count of prevalent cases (vs proportion of background population in primary analysis). Using total prevalent count resulted in a similar prevalence estimate, with a forecast 01/08/2028 prevalence of 993/100 000 (95% CI 956 to 1031) for all IBD. Lastly, by simply applying the annual percentage change from Poisson regression between 2008 and 2018 to 2028, a similar estimated IBD prevalence was reported as in the primary analysis (1252 95% CI 1151 to 1362/100 000).

## Discussion

We describe a comprehensive prevalence analysis of IBD in a defined geographical area of Scotland supporting approximately 900 000 people. Data capture–recapture techniques show that pathology coding was the most accurate information stream in identifying true positives in the prevalent population and overall. However, a lack of significant dependence between information streams supports an approach to prevalence measurement that uses multiple information sources. This has important ramifications for previous studies that have relied on in-patient coding alone, as this underestimated prevalence in our cohort by 47%. We show that it is possible in our cohort to identify 95% of true positives with only 6% false positives using the combination of primary care prescribing of mesalazine, inpatient ICD-10 K50/51 coding and histopathology sources.

A recent nationwide historic, and future-predicted, prevalence IBD analysis has been published in the Canadian population. They report an estimated prevalence of 0.7% in 2018, forecast to rise to 1% of Canadians by 2030.^2^ We report a similar point prevalence of 0.78% on 31/08/2018 but this may be as high as 0.83% when missed cases from capture–recapture analysis are included. This surpasses the most recent UK prevalence estimates in 2003 of 0.4% (equivalent to 221 000 of the 2003 UK population) and that of >0.3% (range 0.27% (Southern Europe) to 0.77% (Northern Europe)) from recent systematic review of North America and Europe prevalence.^2 4^ We report some of the highest incidence of UC, CD and IBDU in the literature. IBDU incidence in particular was high at 5.4/100 000 in 2018. However, IBDU incident and prevalent cases as a proportion of all IBD was unchanged over the 10-year study period, suggesting IBDU is not increasing in our cohort.

Compound prevalence is the phenomenon whereby incidence exceeds mortality, such that prevalence inexorably increases. Western populations with chronic conditions of stable incidence and low mortality are most sensitive to this, which accompanied with the ageing population has important considerations for healthcare provision. In IBD, this has several important nuances. For example, the need for increased use of immunomodulator drugs in the elderly, whom may have more frequent hospitalisations, greater postoperative mortality and in particular thromboembolic risk compared with younger patients.^5 8^

While the overall direct healthcare costs for IBD are forecast to rise (€161 to €661 million between 2011 and 2040 in the Netherlands), current estimates suggest IBD patients ≥60 years may account for only 1% of this.^9^ This may reflect a low level of anti-TNF use (25.3% vs 13.1%, p<0.001 in <60 years vs ≥60 years, respectively), with anti-integrin use not reported.^9^ It is likely that increased use of JAK inhibitors, anti-integrins and access to biosimilar anti-TNF will only increase the >60 years share of total IBD cost, thus future studies to assess health-economic effects of compound prevalence in IBD are needed.

The suggested use of immunomodulators to reduce faecal biomarkers irrespective of symptoms^10^ will have important ramifications for an IBD population of the future whom are mostly >50 years old. For example, recent ECCO practice position urges careful consideration for thiopurine use in the elderly^11^ (HR for lymphoma 5.3, absolute risk 1 in 300 for >70s), yet anti-TNF monotherapy is associated with immunogenicity.

In addition, the median age of patients in ACCENT-1, SONIC and CHARM trials were all <40 years (IQR <15 years), with no patients enrolled >80 years of age (eldest patient in SONIC combination therapy arm was 68 years).^12–14^ The efficacy of IBD treatments in controlled trials of the future should include a distribution of ages representative of our population, not least due to increasing polypharmacy (and therefore drug–drug interactions) and immunosenescence within the elderly.^15 16^

The strengths of the current analysis include the manual verification of all cases using multiple information sources, capture–recapture methodology and the reporting of actual prevalence for the past 10 years to guide future estimates. For example, manual review of EHR (including date of diagnosis calculation) ensures that the likelihood for incident cases diagnosed pre-2008 being carried over is highly unlikely. The main weakness is that despite our rigorous efforts to identify IBD patients, cases may have escaped capture from our data sources leading to an underestimation of IBD prevalence. Capture–recapture methods assume a closed population that each capturing stream is independent and that all members have the same probability of being captured. These assumptions are difficult to satisfy completely in epidemiological studies, thus despite our estimation of missed cases, results herein are best interpreted as minimum prevalence rates in all cases.

For example, those patients with an IBD diagnosis made pre-1990 and who had no further contact with any secondary care and no history of immunomodulator nor mesalazine usage would be missed. However, we believe this number will be very small. Similarly, while efforts were made to identify whether patients had left our health board during case review, it is likely that some patients have left the area without our knowledge. However, we believe that this will be augmented by the overall net migration within Lothian (0.9% of total 1.07% population change between 2016 and 2017 was migration) of which 73% were <34 years of age.

The increased projected healthcare burden of IBD in the next 10 years is set against significant predicted challenges to the NHS over the same period. The UK had among the lowest number of gastroenterologists per head of population in 2007 (1.4 vs 3.5 in France and 3.9 in the USA per 100 000).^17^ This has increased to 2.3/100 000 (https://www.bsg.org.uk/resource/workforce-report-2018.html), yet resources remain scarce to manage IBD patients presently, or the increases we project over the coming 10 years. Innovative strategies for IBD care delivery are therefore required. These are likely to include more capacity for acute IBD flare review, distance monitoring of disease through home calprotectin and diversification of the physician–patient interaction through e-health and videoconferencing solutions.

In summary, we report detailed multiparameter information capture–recapture techniques to describe the prevalence of IBD in Lothian, Scotland. We show 1 in 125 currently have IBD, and this is forecast to rise to 1 in 98 within 10 years. The IBD physician of the future will need to be attuned to the ageing demographic of their patients, with important implications for treatment decisions and healthcare provision.
