# Supplementary figures and images for: IBD prevalence in Lothian, Scotland, derived by capture–recapture methodology
Source: Gut. 2019 Jul 11;68(11):1953–60. doi: 10.1136/gutjnl-2019-318936 (PMC6839733; doi:10.1136/gutjnl-2019-318936)

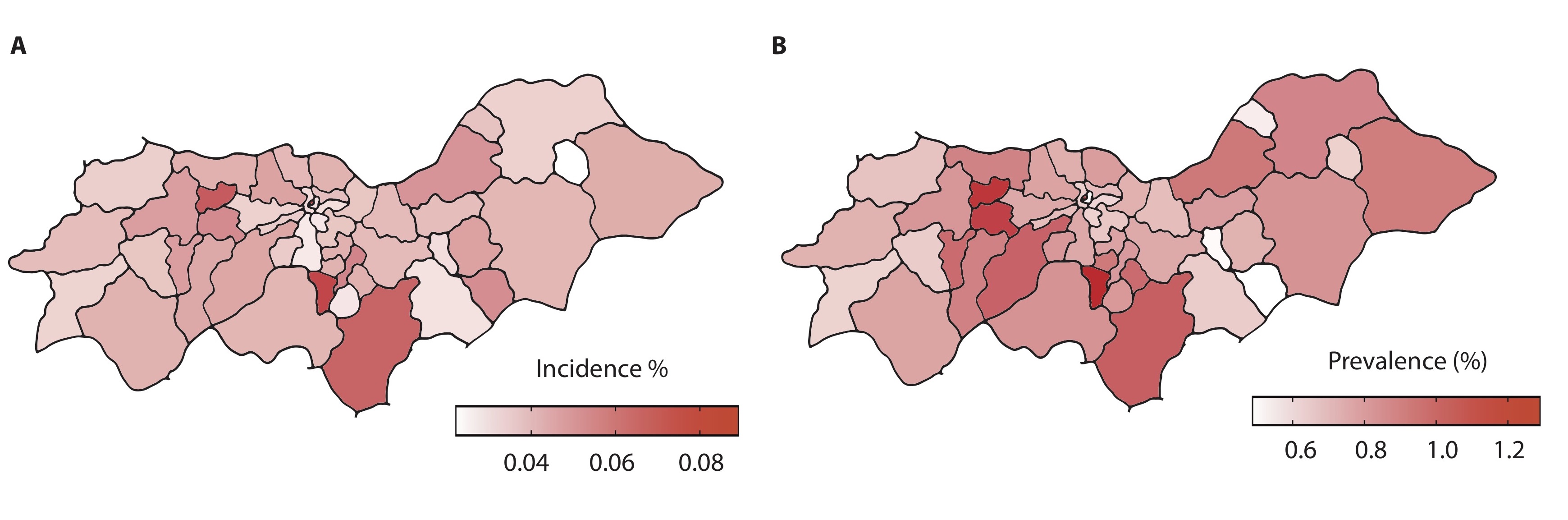

Supplement: Supplementary data [file gutjnl-2019-318936supp001.jpg]

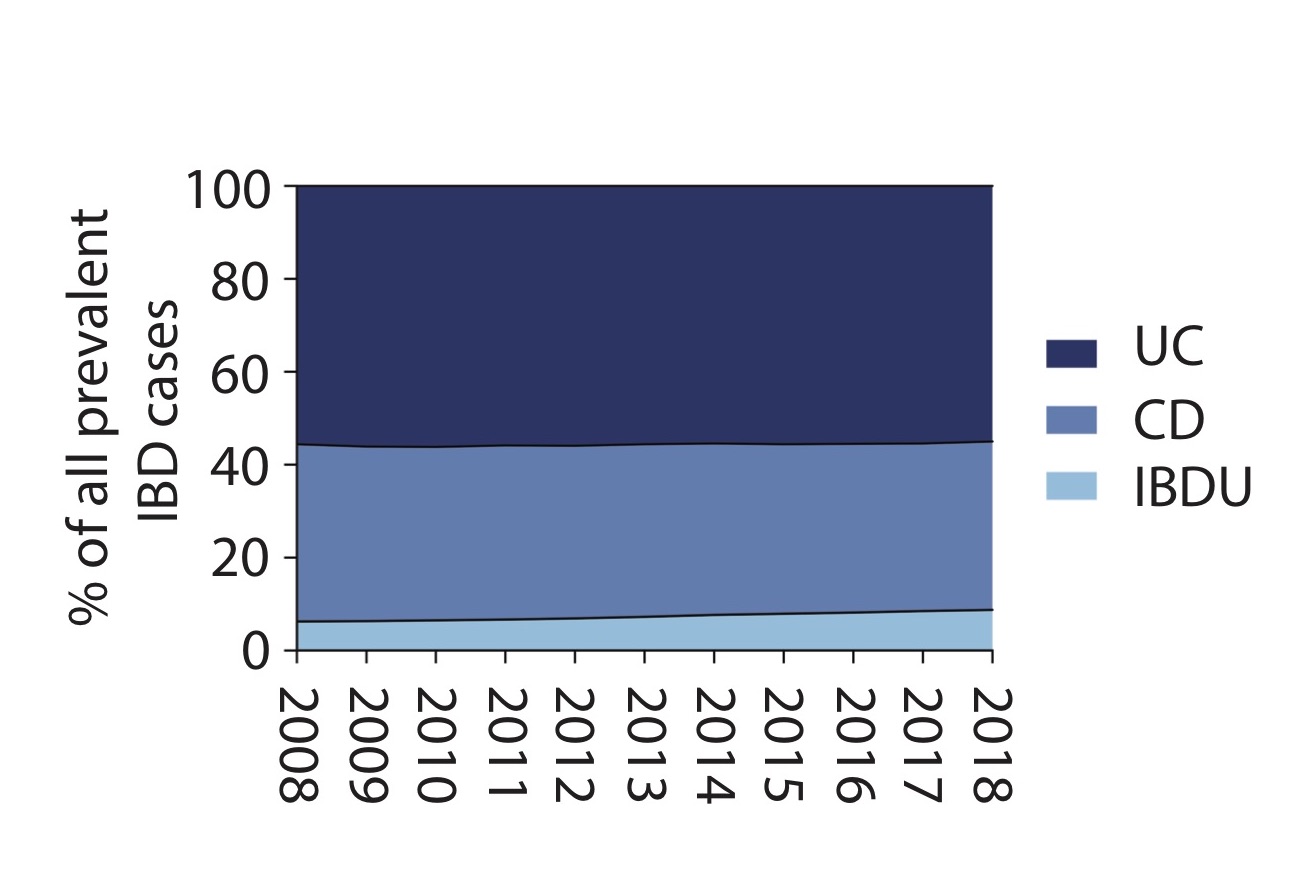

Supplement: Supplementary data [file gutjnl-2019-318936supp003.jpg]

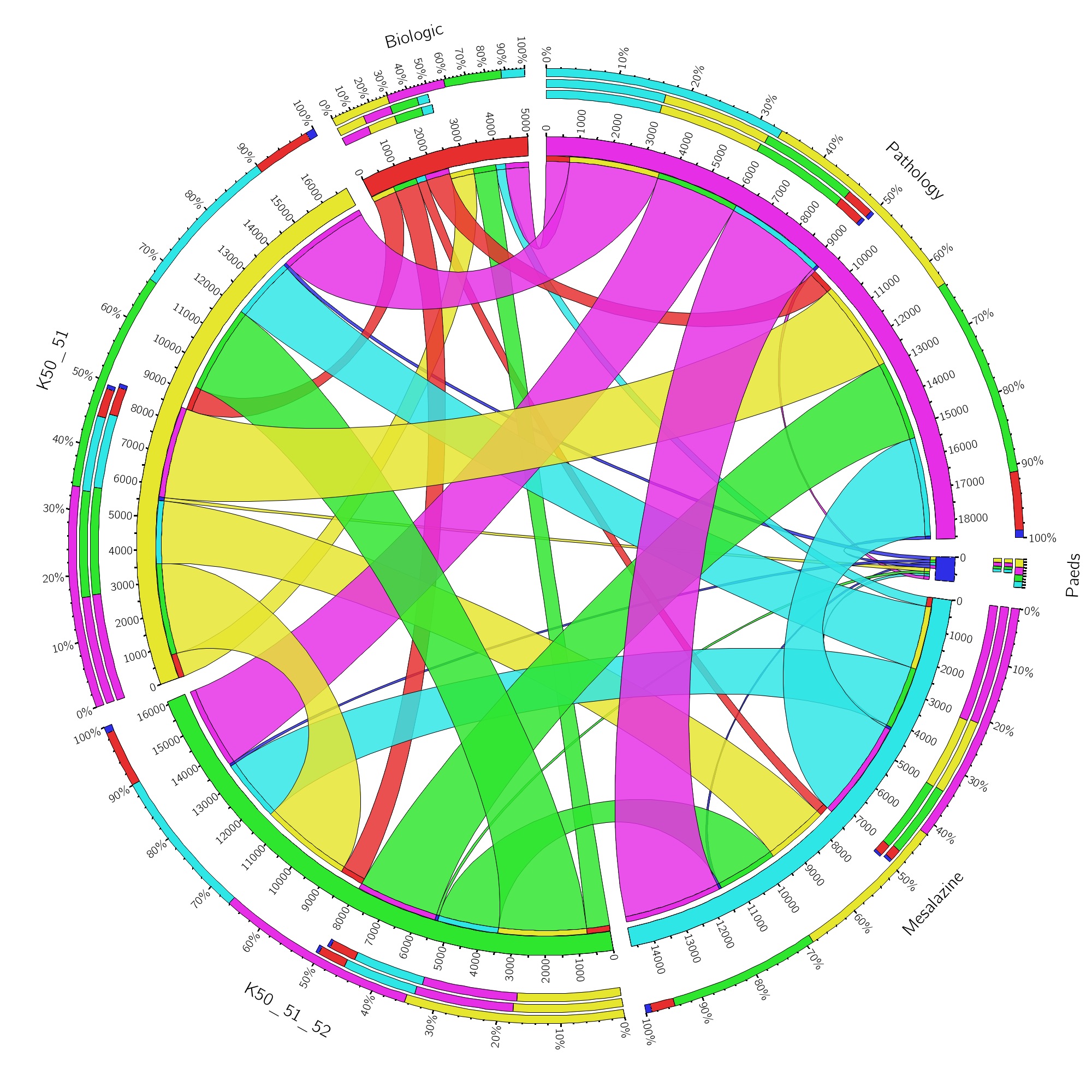

Supplement: Supplementary data [file gutjnl-2019-318936supp004.jpg]

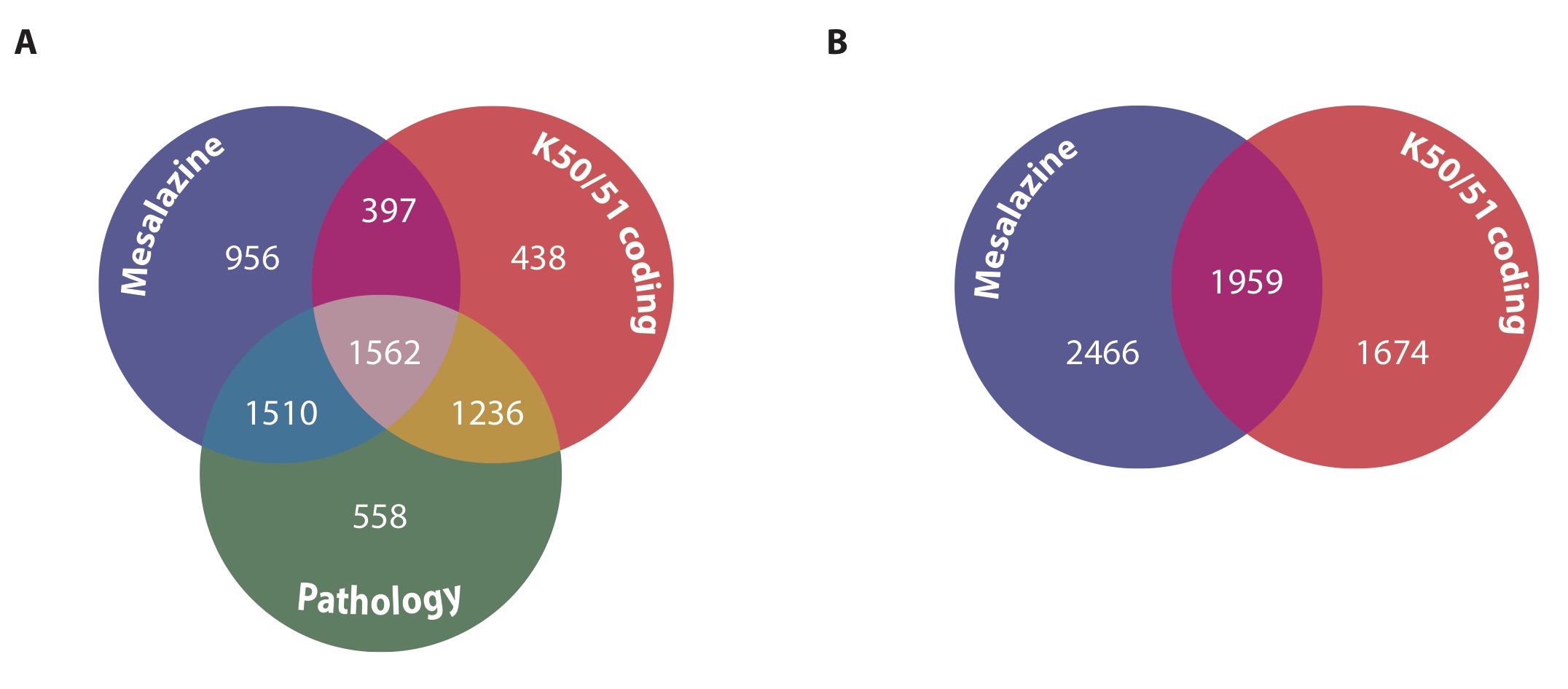

Supplement: Supplementary data [file gutjnl-2019-318936supp005.jpg]
